# Spinal Muscular Atrophy and Communicating Hydrocephalus: A Novel or a Well-Established Rare Association?

**DOI:** 10.7759/cureus.11433

**Published:** 2020-11-11

**Authors:** Fatimah Z Alkhars, Nabil Almajhad, Jaafer Al-Obaid, Fatimah Alghadeer, Ahmed Y Bo Ali

**Affiliations:** 1 Pediatric Department, Maternity and Children’s Hospital, Al-Ahsa, SAU; 2 Radiology Department, King Fahad Hospital, Al-Ahsa, SAU; 3 Pediatrics, College of Medicine, Imam Abdulrahman Bin Faisal University, Dammam, SAU

**Keywords:** spinal muscular atrophy, sma, neuromuscular, blake’s pouch cyst, tetraventricular hydrocephalus

## Abstract

Spinal muscular atrophy (SMA) is a genetic progressive neuromuscular disease characterized by loss of motor neurons, which is linked to mutation of the survival motor neuron-1 gene. Saudi Arabia has a higher than the worldwide prevalence of the disease, estimated to be 4.42/100,000 cases. Association of spinal muscular atrophy with tetraventricular hydrocephalus secondary to Blake’s pouch cyst have rarely been reported. Herein, we report a rare case of genetically confirmed type I spinal muscular atrophy accompanied by communicating hydrocephalus with atypical Blake’s pouch cyst. Further studies are needed to confirm the exact genetic correlation.

## Introduction

Spinal muscular atrophy (SMA) is a progressive neuromuscular disease characterized by degeneration of the motor neurons of the spinal cord anterior horn cells. It is caused by mutations in the survival motor neuron-1 (SMN1) gene located in chromosome 5q in most of cases [1]. The worldwide prevalence of SMA is 1-2/100,000 cases; however, in Saudi Arabia, a higher prevalence was observed, which was estimated at 4.42/100,000 cases. According to the age at onset and the disease severity, SMA has three main subtypes: type I (Werdnig-Hoffmann disease), with onset in non-sitters, before 6 months of age; type II, with onset in sitters, at 6-18 months of age; and type III (Kugelberg-Welander disease), with onset in walkers, after the age of 18 months [2]. The most severe form is type I SMA, which is the leading genetic cause of death during infancy [1]. Spinal muscular atrophy variants are characterized by atypical or additional characteristics beyond those comprising the classic SMA clinical picture. Recently, specific disease-modifying treatment has been developed for some neuromuscular diseases. In 2016, the Food and Drug Administration approved nusinersen (Spinraza), an antisense oligonucleotide, to be the first drug used to treat SMA. Moreover, in 2019, onasemnogene abeparvovec (Zolgensma), a one-time gene replacement therapy, was also approved for the treatment of SMA [3,4]. This report describes a rare case of genetically confirmed type I SMA associated with communicating hydrocephalus.

## Case presentation

The patient was a three-month-old female, the second child of healthy parents with consanguineous marriage. She was referred to the pediatric neurology department with a history of weak cry, poor feeding, and progressive peripheral hypotonia. She was born full-term by normal spontaneous vaginal delivery and had no history of neonatal intensive care unit admission. Growth parameters at birth were within the normal range. Prenatal history revealed decreased fetal activity during the third trimester but was negative for polyhydramnios and urinary tract infection. She had a history of two previous admissions secondary to chest infections but did not require mechanical ventilation. She had one healthy brother and negative family history of similar diseases.

The patient was evaluated for the first time at the age of two months. She was awake, alert, with stable vital signs, without dysmorphic features or neurocutaneous stigmata. Growth parameters showed normal weight and length, but large head circumference, as follows: weight, 4 kg (above 15th percentile); length, 52 cm (at 50th percentile); and head circumference, 46 cm (above 99th percentile). Signs of active hydrocephalus were observed, including a bulging anterior fontanelle, sunset eyes, and dilated scalp veins. Occasional tongue fasciculation was seen. The patient was hypotonic, with flickery movements of the upper limbs and no movements in the lower limbs. Deep tendon reflexes were absent. Apart from paradoxical breathing, the systemic examination was unremarkable with no organomegaly noted.

**Figure 1:**
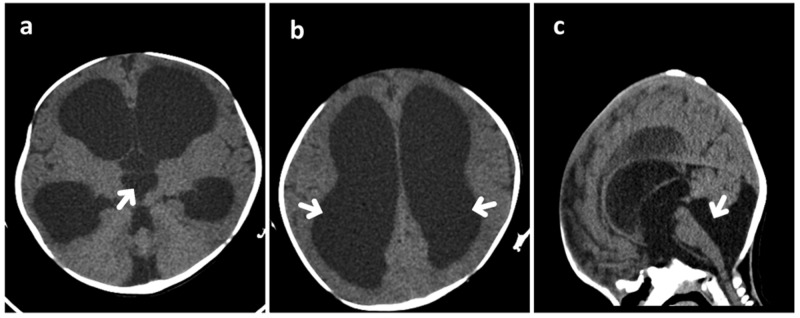
Non-enhanced computed tomography images (NECT) Axial (a,b) and sagittal (c) scans showed marked dilation of the lateral and third ventricles, mildly dilated fourth ventricle, stretching of corpus callosum, and bulging and widening of the anterior fontanelle. The cerebral aqueduct was open and communicated with the cisterna magna. Prominent infravermian cerebrospinal fluid space; however, there was a normally developed cerebellar vermis.

Type I SMA was suspected and molecular genetic testing was requested. Regarding macrocephaly, computer tomography (CT) was done and it showed picture of active communicating hydrocephalus (Figure 1). Tumors, infection, and bleeding were ruled out as possible causes of the hydrocephalus and the patient was referred for urgent surgery. Later on, the molecular genetic study confirmed type I SMA, revealing a homozygous pathogenic deletion encompassing exons 7 and 8 of the SMN1 and extending to the neuronal apoptosis inhibitory protein (NAIP) gene, with two copies of the SMN2 gene.

A ventriculoperitoneal shunt (VPS) was surgically implanted. Nusinersen treatment was planned and requested.

The patient was followed up by a multidisciplinary team comprising neurology, neurosurgery, genetic, pulmonary, and physiotherapy specialists. However, she died at the age of five months secondary to aspiration pneumonia, prior to receiving the first dose of nusinersen.

## Discussion

Spinal muscular atrophy is an autosomal recessive neuromuscular disease related to SMN gene mutation in the majority of cases [4]. Associations between SMA and central nervous system disorders have rarely been reported [5]. Scapuloperoneal SMA, SMA with pontocerebellar hypoplasia, and SMA with progressive myoclonic epilepsy are some of the SMA variants that have been reported in the literature [6]. Herein, we report a case of genetically confirmed type I SMA associated with communicating hydrocephalus. Our patient underwent the initial laboratory work-up including complete blood count, electrolyte levels, renal function tests, liver function tests, and creatine kinase level, which were all within the reference ranges. Additionally, blood ammonia and lactate levels, tandem mass spectroscopy, urinalysis for organic acid, TORCH panel test, echocardiography, and abdominal ultrasound were also performed to rule out possible associations and all had normal findings. To the best of our knowledge, only two reports have suggested an association between type I SMA and tetraventricular hydrocephalus secondary to typical Blake’s pouch cyst (BPC) [5,7]. Shohoud et al. first reported such an association in 2014, and suggested that further studies are needed to identify the genetic link between type I SMA and BPC [8]. Tozawa et al. reported another case of type I SMA complicated by a typical BPC and linked it to the severity of the reduction in the SMN protein levels [5,7]. Blake’s pouch is an embryonic structure arising from the roof of the fourth ventricle posterior membranous area that communicates with the subarachnoid space. When it fails to perforate to form the foramen of Magendie, BPC develops [9]. Typically, BPC is identified by brain magnetic resonance imaging (MRI) and characterized by tetraventricular hydrocephalus, infracerebellar cyst, well-developed non-rotated cerebellar vermis, cystic dilation of the fourth ventricle without cisternal communication, and some compression over the medial cerebellar hemispheres [8]. In our case, however, CT revealed a mildly dilated fourth ventricle and an open cerebral aqueduct communicating with cisterna magna, findings which differ from those in the previously reported cases and the classical picture of BPC. Although CT is insufficient to identify more specific findings, unfortunately, our patient passed away before MRI was performed. 

## Conclusions

In conclusion, this case emphasizes the rare association between type I SMA and communicating hydrocephalus. We recommend that further studies are performed to identify the exact genetic relation. Further experience is also needed to establish the safety, bioviability and efficacy of nusinersen treatment in patients with SMA with an established VPS.
